# A Comparative Study on Micro Hardness and Structural Changes of Dentin Floor Cavity Prepared by Er: YAG Laser Irradiation and Mechanical Bur

**Published:** 2013-06

**Authors:** F Arbabzadeh, E Birang, R Nazem, M Abbasian, F Koosha, R Birang

**Affiliations:** a Dental Materials Research Center, Dept. of Operative Dentistry, Isfahan University of Medical Sciences, Isfahan, Iran.; b Dental Student, Faculty of Dentistry, Shahid Sadoughi University of Medical Sciences, Yazd, Iran.; c Torabinejad Dental Research Center, Dept. of Operative Dentistry, Isfahan University of Medical Sciences, Isfahan, Iran.; d Dentist, School of Dentistry, Isfahan University of Medical Science, Isfahan, Iran.; e Dental Student Research Center, School of Dentistry, Isfahan University of Medical Sciences, Isfahan, Iran.; f Dental Implant Research Center, Dept. of Periodontics, School of Dentistry, Isfahan University of Medical Sciences, Isfahan, Iran.

**Keywords:** Vickers Hardness Test, Bur, Er: YAG laser, Microhardness

## Abstract

**Statement of Problem:** Laser irradiation makes structural and chemical changes on the dental hard tissues. These changes alter the level of solubility and permeability of dentin.

**Purpose:** The aim of this study was to compare the microhardness and the structural changes in the dentin cavity floor prepared with Er: YAG laser and bur.

**Material and Methods: **In this experimental study, fifteen intact human molars were selected. Two square cavities were prepared on the buccal and lingual surfaces of each tooth. One side was randomly prepared by Er:YAG laser and the other side by bur. The specimens were divided into two halves. Consequently, there were 30 samples in every group. One half was assigned for the Vickers’s hardness test and the other one, for determination of Ca and P percentage and atomic elements analysis. The data were analyzed by Paired T-tests through SPSS16 (α≤o.o5).

**Results: **The means and the standard deviation of the microhardness were 69.77±25.62 and 51.33±9.31 Kg/mm^2^ in the laser and bur groups, respectively. Statistical analysis showed significant differences between the two groups (*p*=0.017). Weight percentage of calcium in the laser cavity (65.5) was less than the bur cavities (68.21) and the difference was significant (*p*= 0.037).

**Conclusion: **The hardness of dentin in laser group was higher than the bur group because of the higher mineral content of the dentin. The hardness and the mineral content of dentin are important factors in the bonding effectiveness of the dental materials so with laser cavity preparation, good mineral substrate are available for a better bonding.

## Introduction

 By introduction of the ruby ​​laser by Maiman in 1960, different types of lasers in dental field were gradually introduced. One of the purposes of laser in dentistry is caries removal and preparation of hard tissue, but the exposure of hard tissue with lasers such as CO₂, Neodymium- Yttrium Aluminum Garnet (Nd:YAG) needs high energy density and has side effects such as melting, carbonization, defecting the surroundings tissue and increasing the pulp temperature [1-3]. To reduce the side effects of high intensity lasers; Erbium laser family (i.e., Er: YAG, and Er Cr: YSGG) were introduced. The Er: YAG laser with a wavelength close to the maximum water uptake (2940 nm) and with least amount of side effects has the most cutting efficacy. The hard tissue removal and the cavity preparation with Er: YAG laser causes chemical and morphological changes in dentin left at the floor of cavity. The rate of the changes is associated with the absorption characteristics of the target and the radiation parameters such as frequency, radiation energy, time and mode of radiation exposure [4].

Nevertheless, many studies showed that Er:YAG laser was a good substitute for the mechanical cutting tools such as burs and cavity preparation with minimal effect on the healthy tissue, teeth and surrounding tissues. When comparison to the rotary equipment, although cavity preparation with Er: YAG laser needs more times. However, the benefits of laser such as having less noise, no vibration and no need for the anesthesia application are the significant points especially in pedodontics [5-6].

Previous studies have compared the remaining dentin in the cavity floor prepared by Er: YAG laser and diamond burs in two methods [5-6]. 

The first method was measuring the micro hardness of the cavities. The microhardness of the dentin of the cavity floor is measured by Vickers method.

The microhardness of the dentin represents the quantity of​​ calcified mineral matrix in a square millimeter of dentin [1].

 The second method was through the atomic energy dispersive X-ray spectroscopy (SEM-EDX - scanning electron microscopes) [7]. This device calculates the percentage of different elements in each level through radiating the target surface and analysis of reflected waves [5]. The studies carried out with Er: YAG laser using the same parameters resulted in conflicting findings. In a study conducted by Celik et al. [4], dentin microhardness and percentage of calcium and phosphor elements in the laser and bur cavities were similar, but in study of Hossain et al. [8], mineral elements after laser irradiation significantly increased. In another study, conducted by Souza Gabriel et al. [9], laser with similar parameters increased dentin microhardness.

Morphological characteristics of the dentin surfaces play an important role in the stability of restoration. Micrographs, obtained by electron microscopes, showed that the dentin surface, irradiated by Er: YAG laser, had become rough and had more irregularities for sufficient retention of tooth-colored restorative materials [8]. In addition, smear layer had been removed and the dentinal tubules had become opened [10] because of having more water composition [11-12]. Therefore, the dentin surface, prepared by Er: YAG laser, in comparison with bur, has more peritubular dentin, and the zone between peritubular and intertubular dentin is more prominent [13].

The aim of this study was to compare the microhardness and the structural changes in the dentin of the cavity floor prepared by Er: YAG laser and bur.

## Material and Methods

In this experimental study, fifteen intact human molars were selected and preserved in thymol solution 0.1% for a week. Thymol solution has the antibacterial effect and does not change the dentin composition .These teeth were either third molar teeth or had been extracted for an orthodontic purpose. Then, the specimens were stored in distilled water for a month at 4˚̊C. For more convenience, samples were mounted from the apex to the CEJ in the acrylic block (Acropars; Tehran, Iran). After preparing the samples, on the buccal and lingual surfaces of each tooth, a square was drawn with dimensions of 3 ×3 mm. Finally, 30 squares were obtained. Then, 15 squares were randomly selected as the control group. In this group, we used the dental turbine and fissure bur ¼ (Teezkavan; Tehran, Iran,) for preparation of cavities with a depth of 2 mm, cleaned with water spray and dried with air. The other 15 squares remained as the test group and underwent the same process using Er: YAG laser with wave length of 2940nm (Fotona; Fidelis plus, Ljubljana, Slovenia,) energy 1watt 250mj, 4Hz, Short Pulse Mode (SPmode) and pulse duration of 250μs [4]. The hand piece used in this study was RO7 with contact mode of air and water spray (50 percent each one). Cavities were measured with periodontal probe with the same depth; 2mm, which was the same for the entire molars .Every specimen was divided into two halves (mesial and distal) with a low speed saw (Isomet; Buehler Ltd, Illinois, USA) in both groups. Therefore, there were 30 samples (halves) in every group. One half was assigned for Vickers hardness test and the other half for determination of the percentage of Ca and P and atomic elements analysis. The samples selected for atomic elements analysis, were extracted by ethanol with different concentrations and were then carbon coated. Ten samples out of the fifteen were accomplished for this test. Atomic analysis was conducted by SEM-EDX (Seron AIS2003 Gyeonggi-do; Korea). The Vickers measurement of microhardness was conducted on three points in a straight line in depth of 30 micron from the bottom of cavities. For a blind statistical evaluation of the microhardness and the atomic analysis, lasers cavities were marked with a cross and bur cavities with a circle. The data were analyzed by SPSS (ver. 16) through Paired T-tests.

## Results

The findings of the microhardness measurements by Vickers method have been presented in table 1. 

**Table 1:** Vickers hardness test: numbers of the laser and the bur cavities and their means

**Laser**				**Bur**			
**Vickers hardness**			**Mean**	**Vickers hardness**			**Mean**
62.60	90.3	63.60	70.03	46.10	45.30	44.70	45.37
62.60	51.50	71.30	61.80	41.30	46.70	49.20	45.73
59.10	87.60	61.30	69.33	43.90	44.70	42.60	43.73
55.10	62.60	68.60	62.10	47.90	44.70	46.70	46.43
57.10	59.10	60	58.73	47.60	55.10	48.20	50.30
60.40	66	73	66.47	97.50	89.90	56.70	81.37
73.60	61.70	66	67.10	48.20	54.40	55.30	52.63
157	170	157	161.33	48.20	56.30	53.30	52.60
63.60	63.60	62.60	63.27	47.20	49.20	54.40	59.27
59.10	49.80	60.40	56.43	46.70	47.60	57.10	50.47
62.30	58.20	67.50	62.67	49.20	41.50	44.80	45.17
64.50	73	57.80	65.10	50	51.20	53.60	51.60
55	62.40	68.50	61.97	47.80	44.60	46.60	46.33
62	51.40	71.20	61.53	41.20	46.50	49.10	45.60
57.20	59	59.90	58.70	47.70	55.20	48.30	50.40

**Figure 1:**
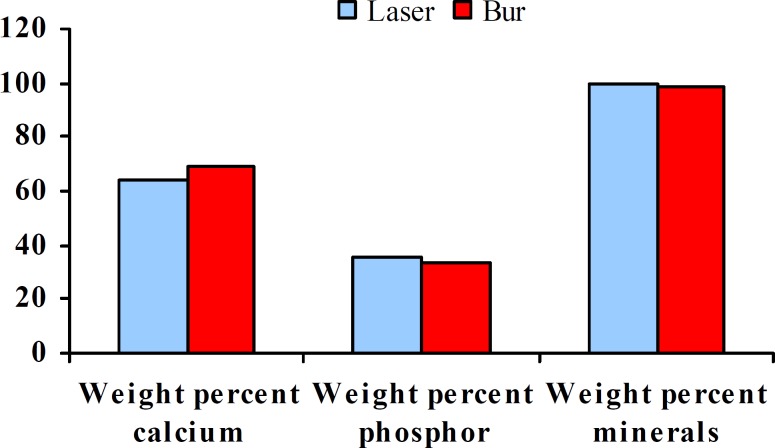
Comparison of the weight percent of calcium, phosphorus in the two methods of laser and bur

The Means and the standard deviations of the microhardness were 69.77±25.62 and 51.33±9.31 in the laser and bur cavities, respectively. Statistical analysis showed significant differences between two groups (*p*= 0.017). The results of the measurements of calcium and phosphorus in the cavities have been shown in Table 2. Mean scores of atomic analysis showed that the atomic weight percentage of phosphorus in the laser cavity (34.48) was more than the bur cavity (31.28), and this difference was significant (*p*= 0.035). Weight percentage of calcium in the laser cavity (65.5) was less than the bur cavities (68.21), the difference was significant (*p*= 0.037) (Figure 1). In the statistical analysis, a significant differences was seen between the calcium- phosphate ratio of the laser and the bur cavities (*p*= 0.036).

## Discussion

**Microhardness**

The present study showed that the microhardness of dentin in cavity preparation by the Er:YAG laser with parameters of energy 1watt/ 250mJ 4Hz with pulse duration of 250µs was significantly higher than the cavities prepared by bur. As the hardness of dentin indicates the amount of its calcified matrix; the dentin which is irradiated by laser would have a higher resistance to acid etching [17].

Similar results were obtained by Souza-Gabriel et al. study conducted in 2009[9]. In that study, hardness of cavity dentin prepared with Er: YAG laser with similar parameters of our study was higher than the bur cut dentin [9], but in study of Celik et al., with the same parameters as our study, there were no significant differences in hardness between the cavities prepared with bur and laser. The inconsistency between Celiks’ study and our study could be due to the depth of the hardness measurement. This depth was 50 microns from the cavity floor in Celik’s study but 30 microns in our study. It is obvious that more distance from the cavity floor limits the laser exposure [4]. However, Koramota et al. [18] in 2001 reported that laser irradiation with high energy density significantly reduces the microhardness of the hard tissue. This condition has been named among unsuitable characteristic of dental hard tissue for bonding restorations.

**Table 2:** Weight percent of calcium and phosphor in laser and bur cavities

**Laser cavities**			**Bur cavities**		
**Weight percent of** **calcium**	**Weight percent of** **phosphor**	**Ca/ P ratio**	**Weight percent of** **calcium**	**Weight percent of** **phosphor**	**Ca/ P ratio**
35.47	64.53	1.18	68.95	31.05	2.22
36.04	63.96	1.77	66.69	33.31	2
30.89	69.11	2.23	72	28	2.57
32.65	67.35	2.032	62.35	36.65	1.72
37.2	62.8	1.68	66.66	33.35	1.81
36.11	63.89	1.76	73.12	26.88	2.71
35.11	64.89	1.84	67.90	32.10	2.11
34.69	65.31	1.88	64.42	35.58	1.81
32.35	67.65	2.09	72.08	22.92	3.36
34.33	65.68	1.91	67.01	32.99	2.03

**Atomic analysis with SEM-EDX**

In modern operative dentistry, bonding systems are used to increase bond strength between dentin adhesive systems and restorative material to prevent leakage (micro leakage) around the edges of the restoration so that reducing the risk of secondary caries. These bonding systems must have low surface tension, and the dentin and the restorative material should have high surface energy to attain maximum contact surface energy [19-20]. With regard to the surface energy, hydroxyapatite has high surface energy and collagen has a low surface energy while overall surface energy of dentin is generally low [21].

Since irradiation with Er: YAG laser leads to vaporization of the organic material in dentin; remained mineral component (hydroxyapatite) has a higher surface energy balance. Microscopic studies have also shown that laser irradiation causes many surface irregularities in micron diameters. Therefore, laser improves chemical and mechanical bonding strength between dentin and restorative materials through altering the dentin surface with higher surface energy and more irregularities. Although dentin is a hard and mineral tissue, it is also flexible and supports the brittle enamel [8]. The increase of calcium leads to chalkiness and rigidity of the dentin which is in juxtapose to the natural characteristic of the dentin. Therefore, changes in the ratio of Ca / P can be positively effective in the restoration quality [7]. The results of the present study showed that the amount of mineral component in dentin, prepared by using laser, is significantly more than dentin prepared with bur.

Significant amount of phosphor in the dentin of the cavity prepared with laser has a positive effect in increasing the surface energy of dentin. Meanwhile, the weight percent of calcium was less in the laser- prepared cavity compared to bur cavities in our study, which prevents the dentin excessive rigidity. These results concord with the results of a study conducted by Shahabi et al. [7] with Er, Cr: YSGG laser, and with the same parameters of our study indicating similar specifications of erbium laser family (i.e., Er: YAG, and Er Cr: YSGG) [5]. In a similar study, conducted by EU Celik et al. [4] with Er, Cr: YSGG laser, there was no significant difference in calcium and phosphor percentage between cavities prepared with bur and laser. In that study, enamel was removed by using the high intensity laser, but dentin was removed by laser with the same parameters of our study. Hossain et al. [8], in their study showed that the P, Ca percentage in the laser cavity was significantly higher than the bur- prepared cavities; the evaluation of the laser-prepared cavity with an electron microscope showed an irregular surface with no smear layer and completely open dentinal tubules.

Further subsequent studies can investigate laser irradiation with different parameters, and burs with different cut surfaces. The properties of dentin cut with different laser parameters can be compared with the dentin cut by optimum condition. One of the limitations of the present study was the long time taken for the laser cavity preparation.

## Conclusion

Based on the results of this study, the hardness of the dentin in the laser- prepared cavities was higher than the bur- prepared cavities because of the higher mineral content of dentin in laser- prepared cavities. Laser- prepared cavities contain less calcium and more phosphor when compared to the cavities prepared by bur. Therefore, calcium-phosphorus ratio was less in the laser- prepared cavities compared to bur- prepared cavities. The hardness and the mineral content of dentin are important factors in the bonding to the dental materials so in a cavity prepared by laser, a better bonding is expected.
